# Enhancing Port Performance through Digital Transformation: The Role of Networking Capability and Transformational Organization in Indonesian Container Ports

**DOI:** 10.12688/f1000research.176261.1

**Published:** 2026-01-20

**Authors:** Buyung Pramitra, Mochammad Al Musadieq, Tri Wulida Afrianty, Teuku Noerman

**Affiliations:** 1 Faculty of Administrative Science, Brawijaya University, Malang, East Java, Indonesia

**Keywords:** Port Performance; Digital Transformation; Networking Capability; Transfor-mational Organization; Indonesian Container Ports

## Abstract

This study aims to examine the influence of networking capabilities and transformational organizational characteristics on digital adoption, as well as its impact on port performance at container ports in Indonesia. This study also analyzes the mediating role of digital adoption in the relationship between organizational capabilities and port performance. The study uses an explanatory design with a quantitative approach through a survey method. Data were collected using structured questionnaires from 103 container ports throughout Indonesia with respondents being decision makers and core port function managers, namely General Managers or Operations Managers or Marketing Manag-ers if the General Manager was not available. Data analysis was conducted using Structural Equation Modeling with a Partial Least Squares approach through SmartPLS. The results of the study indicate that networking and transformational organizational capabilities have a significant effect on digital adoption, and digital adoption has a significant effect on port performance. This study also found that digital adoption significantly mediates the relationship between transformational organization and port performance. However, this study does not prove that digital adoption mediates the influence of net-working capability on port performance.

## 1. Introduction

The global maritime industry is undergoing rapid transformation driven by increasing international trade volumes, evolving customer expectations, and technological advancements. ^1^ Container ports, as critical nodes in the global supply chain, play a pivotal role in ensuring the smooth and efficient movement of goods. ^2^ The operational performance of these ports directly affects logistics costs, delivery times, and overall economic competitiveness of countries. ^3^ In this context, the adoption of digital technologies has emerged as a key enabler for ports to enhance operational efficiency, optimize resource allocation, and improve service quality. ^2, 4^

Digital transformation in ports involves the integration of advanced technologies such as automation systems, Internet of Things (IoT), big data analytics, and blockchain to streamline cargo handling, improve decision-making processes, and enhance transparency. ^5^ While the benefits of digital adoption are widely recognized, the success of such initiatives heavily depends on the organizational capabilities and leadership driving the transformation. ^6^ Networking capability, defined as an organization’s ability to establish, manage, and utilize external relationships, facilitates access to knowledge, technology, and resources necessary for digital innovation. ^7, 8^ Meanwhile, transformational organizations characterized by visionary leadership, adaptive culture, and employee empowerment create conducive environments for embracing change and technological advancements. ^9^

Several studies have explored the impact of digital transformation on port efficiency and competitiveness. Li et al. ^10^ emphasize that digital Transformation enhance Sustainable development of port performance. Prior research indicates that organizations embedded in strong external networks or innovation ecosystems are more likely to achieve successful technology adoption and enhanced innovation outcomes, as these networks facilitate access to external knowledge, collaboration opportunities, and resource integration. ^11, 12^ However, the majority of these studies focus on developed countries with advanced port infrastructures, often overlooking the distinct challenges faced by ports in developing economies.

Indonesia, as the world’s largest archipelagic state, depends heavily on maritime transport and container ports to connect its numerous islands and support international trade. ^13^ The country’s container ports are regulated and classified according to standards such as BCH and BSH, as stipulated in the Director General of Sea Transportation Regulation No. HK.103/2/18/DJPL-16 Year 2016, which categorizes ports into classes A, B, C, and D based on operational capacity and performance. Despite the strategic importance of these ports, Indonesia faces several obstacles in fully leveraging digital transformation, including fragmented infrastructure, inconsistent regulatory enforcement, and limited organizational readiness for change.

This situation creates a critical research gap regarding how internal organizational factors like networking capability and transformational organizational characteristics influence digital adoption, and ultimately, port performance in Indonesia’s container port sector. ^14^ Few empirical studies investigate these relationships in emerging market contexts, where resource constraints and institutional complexities differ markedly from developed economies. ^15, 16^ Addressing this gap will not only enrich the academic understanding of digital transformation in port management but also provide practical guidance tailored to Indonesia’s unique operational environment. ^17^

The novelty of this research lies in its holistic approach that integrates organizational theory with digital innovation studies within the Indonesian container port setting. By examining the mediating role of digital adoption between organizational capabilities and port performance, this study advances current knowledge by highlighting the mechanisms through which digital transformation contributes to operational excellence in a developing country context.

The main objectives of this study are to examine the influence of networking capability on digital adoption in Indonesian container ports, to investigate the effect of transformational organizational characteristics on digital adoption, and to evaluate the impact of digital adoption on port performance. In addition, this study aims to explore the mediating role of digital adoption in linking networking capability and transformational organization to port performance.

Focusing on container ports in Indonesia is essential because of their central role in national trade and the challenges they face in digital integration compared to ports in developed countries. Insights from this research will offer valuable implications for policymakers, port authorities, and practitioners striving to enhance port competitiveness through targeted investments in organizational capacity building and digital infrastructure.

## 2. Literature review

The literature review constitutes the theoretical foundation of this study by critically examining existing research related to the key constructs: networking capability, trans-formational organization, digital adoption, and port performance. This review provides a comprehensive understanding of prior studies, identifies gaps or limitations, and establishes the rationale for the current research.

### 2.1 Networking capability and digital adoption

Networking capability is fundamentally an organization’s ability to build, manage, and utilize relationships with external stakeholders such as partners, suppliers, customers, and regulators. ^18– 20^ This capability facilitates the flow of information, resources, and knowledge essential for innovation and adaptation in dynamic environments. In industries such as maritime, effective networking enables ports to collaborate with multiple actors in the supply chain, aligning operations and sharing best practices, which contributes to operational improvements. ^21^ Prior research shows that networking strengthens trust and coordination among multiple stakeholders, which are essential for effective collaboration in complex operations. ^22– 24^

Networking capability supports continuous learning and adaptation by exposing the organization to diverse perspectives and innovations from the external environment. ^7, 25^ In the context of digital transformation, networking helps overcome internal limitations such as knowledge gaps or resource constraints, making it a critical antecedent to successful digital adoption in ports. ^26^

Networking capabilities enable organizations to gain broader access to information, technological knowledge, and collaboration opportunities that support the digitization process. Waty et al. ^27^ show that networking capabilities have a significant effect on digital adoption and business agility. The findings of Waty et al. ^27^ indicate that organizations with strong networking capabilities are better able to adopt digital technology quickly and effectively, as extensive networks facilitate access to the latest technological innovations and knowledge exchange with business partners. These findings are reinforced by Al Halbusi et al., ^28^ who reveal that social media network capabilities have a significant influence on social media adoption in micro, small, and medium enterprises. Al Halbusi et al. ^28^ research emphasizes that customer engagement and the effectiveness of social networks strengthen the use of social media as a business tool. Overall, both studies show that networking capabilities facilitate the digital adoption process by increasing access to technological resources, strengthening collaboration, and accelerating the digitization process of organizations.

The relationship between networking capabilities and digital adoption can be explained through the perspective of Dynamic Capability Theory by Teece et al. ^29^ In this framework, networking capabilities are viewed as dynamic capabilities that enable organizations to respond to the demands of digitalization. Digital Transformation encompasses the process of integrating digital technology into an organization’s operational and strategic activities to improve efficiency, innovation, and service quality. ^30^ Based on this theoretical foundation and empirical findings, we formulate the following hypothesis:

H1:
*Networking capability has a significant effect on digital adoption.*

### 2.2 Transformational organization and digital adoption

Transformational organization refers to an entity characterized by leadership and cultural attributes that promote change, innovation, and employee engagement. ^9^ Transformational leaders articulate a compelling vision, inspire motivation, and foster an environment where employees are encouraged to embrace change and challenge the status quo. ^31^ Such leadership is critical in digital transformation initiatives, which often encounter resistance due to uncertainty and disruption of established routines. ^32^

Leaders who empower employees and promote learning cultivate a culture that supports experimentation and continuous improvement, both necessary for integrating complex digital systems. ^33^ Furthermore, transformational organizations typically exhibit flexible structures and open communication, which facilitate coordination across departments and with external partners. ^34^ This organizational characteristic not only drives acceptance of digital tools but also ensures their effective utilization by aligning digital strategies with organizational goals and employee capabilities. ^35^ Consequently, transformational organization plays a pivotal role in ensuring that digital adoption translates into meaningful improvements in port performance. ^36^

Research by Kraft et al. ^37^ found that organizational transformation in small and medium-sized enterprises in Switzerland plays an important role in accelerating the adoption of digital tools, such as management software and online platforms. These findings are reinforced by Bunjak et al., ^38^ who emphasize that transformational leadership and shared leadership play an important role in encouraging employees to adopt information technology innovations. A work environment that supports innovation, learning, and change management allows employees to be more open to the use of new technologies. Additionally, Zahra et al. ^39^ highlight that organizational transformation supported by strong leadership and an innovative culture is a key factor in accelerating digital adoption in the industrial sector. Overall, these findings confirm that organizational transformation creates the internal readiness necessary to effectively accept, implement, and utilize digital technology.

From the perspective of Dynamic Capability Theory, organizational transformation can be understood as the ability of an organization to continuously adapt, reconfigure, and develop internal resources and capabilities in response to environmental changes. ^29^ Digital adoption itself encompasses the process of integrating digital technology into an organization’s operational and strategic activities to improve efficiency, innovation, and service quality. ^30^ Therefore, transformational organizations have an advantage in orchestrating the internal changes needed to effectively adopt digital technology. Based on this theoretical foundation and empirical evidence, we formulate the following hypothesis:

H2:
*Transformational Organization has a significant effect on digital adoption.*

### 2.3 Digital adoption and port performance

Digital adoption encompasses the process of integrating digital technologies into an organization’s operational and strategic activities to enhance efficiency, innovation, and service quality. This process is not limited to the acquisition of technology, but also requires changes in organizational processes, skills, and work culture. ^40^ In port operations, digital adoption can be realized through cargo handling automation, real-time tracking, electronic documentation, and the use of data analytics to support decision making. ^17^ These implementations have direct implications for increasing cargo throughput and user satisfaction, which ultimately strengthen port competitiveness. ^41^

Several previous studies have examined the relationship between digital adoption and port performance. Subasinghe, ^42^ through a systematic literature review, concludes that port digitalization plays an important role in enhancing competitiveness and operational sustainability. Technologies such as automation, the Internet of Things (IoT), and artificial intelligence help ports manage cargo and information flows more efficiently, reduce operational costs, and strengthen collaboration among stakeholders. Digitalization also contributes to achieving sustainability targets. Jiang et al. ^43^ find that port-centric information integration has a significant effect on port performance. The adoption of internal and external information systems improves operational efficiency, strengthens coordination among stakeholders, and accelerates data-driven decision making. In addition, Othman et al. ^44^ emphasize that the implementation of smart port practices and advanced technologies supports sustainable port performance by strengthening economic, social, and environmental dimensions. Overall, these findings indicate that digital adoption enhances port performance through improvements in operational efficiency, service quality, competitiveness, and sustainability.

From a theoretical perspective, digital adoption can be understood through the Resource-Based View and Dynamic Capability perspectives, namely as a strategic mechanism that enables ports to optimize and reconfigure their operational resources in alignment with dynamic environmental demands. ^29, 45^ Digital technologies allow ports to improve workflows, accelerate service processes, increase transparency, and strengthen coordination among stakeholders within the port ecosystem. However, the impact of digital adoption on performance is strongly influenced by the readiness of internal organizational capabilities and the external environmental conditions surrounding the port. ^46^ Therefore, understanding the drivers of digital adoption is crucial in order to maximize the benefits of digital transformation. ^47, 48^ Based on the discussion above, the following hypothesis is formulated:

H3:
*Digital Adoption has a significant effect on port performance.*

### 2.4 Mediating role of digital adoption

From a capability perspective, organizational capabilities do not automatically result in better performance if they are not realized through the adoption and utilization of digital technology in business processes. Digital transformation is enabled by organizational and managerial capabilities that help firms identify digital opportunities, reconfigure resources, and embed digital technologies into day-to-day operational processes. ^49, 50^ Heredia et al. ^51^ provide empirical evidence that digital capabilities and organizational readiness contribute to company performance, particularly through digital adoption. This study shows that the benefits of new organizational capabilities can be realized when digital technology is integrated into the operational and strategic activities of the organization. Hadi ^52^ emphasizes that organizational capabilities developed through digital transformation initiatives and strategic leadership influence organizational performance through the digital implementation process. This study highlights that digital initiatives serve as channels that enable organizational capabilities to be operationalized effectively. Therefore, performance improvement can also be influenced by the extent to which digital technology is successfully adopted and aligned with organizational processes.

Second, transformational organizations contribute to digital adoption through the creation of an innovative culture, leadership that supports change, and the strengthening of human resource readiness. Yang et al. ^32^ explain that the combination of access to external knowledge and an innovative culture strengthens the integration and utilization of digital technology. In addition, transformational organizational attributes help overcome adoption barriers such as resistance to change and technical skill limitations, thereby increasing the probability of successful digital transformation. ^53, 54^ When organizations successfully adopt digital technology more effectively, port performance improvements become more likely through process, coordination, and service improvements.

Organizations with strong capabilities tend to perform better, mainly because they are more effective in adopting and utilizing digital solutions. ^55– 57^ Thus, understanding the mediating role of digital adoption is important for port managers because improving performance is not enough just by strengthening organizational capabilities, but must also be accompanied by targeted digital initiatives so that these capabilities truly result in operational improvements. Based on the theoretical arguments and empirical findings above, the research hypothesis is formulated as follows:

H3:
*Digital adoption significantly mediates the relationship between networking capability and port performance.*

H4:
*Digital adoption significantly mediates the relationship between transformational organization and port performance.*

**Figure 1.**
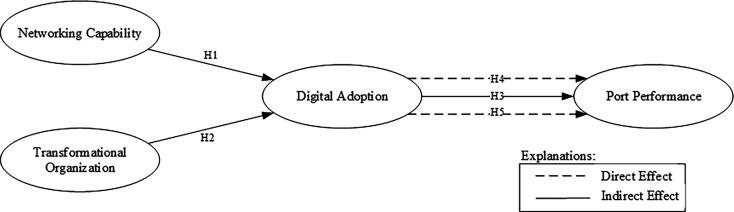
Conceptual framework.

The research model is illustrated in Figure 1.

## 3. Research method

This section describes the research methodology employed in this study in sufficient detail to enable reproducibility. It covers the type of research, research approach, data collection techniques, sampling, and data analysis methods. Based on the complexity level of the problem, research can be categorized into exploratory, descriptive, and explanatory types. ^58^ This study aims to explain the relationships among variables, thus it is categorized as explanatory research. ^59^ Using a positivist or quantitative approach, the study applies deductive reasoning by formulating hypotheses to address the research problems. ^60^ The hypotheses are then empirically tested to verify the underlying theories or concepts, positioning this research as confirmatory in nature. This research employs a quantitative approach with a survey method as the primary data collection technique. Questionnaires were distributed to respondents selected as representatives of the overall population. The questionnaire was designed to measure the research variables, enabling the collection of relevant and accurate data.

Data analysis was conducted using Structural Equation Modeling (SEM) with the Partial Least Squares (PLS) approach. ^61^ SEM-PLS was chosen for its capability to handle complex models and effectively measure latent variable relationships, especially in exploratory and predictive research involving numerous indicators and latent constructs. ^58^ The data processing was performed using SmartPLS version 3, which offers comprehensive features for SEM-PLS analysis. In this study, reflective first-order measurement models were employed, where indicators reflect the underlying latent construct. ^62^ The causal relationship is assumed from the construct to the indicators, which is suitable for measuring perceptions and attitudes of respondents in the context of this research. The research was conducted across container ports throughout Indonesia. The study specifically examines the influence of Networking Capability, and Transformational Organization on Port Performance, with Digital Adoption serving as mediating variables.

The population in this study includes all ports that conduct container handling activities in Indonesia. In the operational context, these ports consist of two categories, namely ports that specifically handle containers and multipurpose ports that also handle containers. The unit of analysis in this study is the organization, namely ports that conduct container handling activities in Indonesia. This study involves 103 container ports as the sample, thus exceeding the minimum threshold and providing an adequate basis for parameter estimation in SEM-PLS. Data collection was conducted using a structured survey through questionnaires delivered to key informants at each port. Respondents were determined at the level of decision-makers and managers of core port functions, with the General Manager (GM) as the primary choice, or the Operations Manager or Marketing Manager if the GM was not available. The selection of key informants aims to ensure that the responses provided accurately reflect organizational conditions, given that the variables examined (Networking Capability, Transformational Organization, Digital Adoption, and Port Performance) are directly related to managerial practices, inter-organizational coordination, and port operational capabilities. In addition, selecting respondents at the managerial level is expected to minimize information bias and improve the quality of data used in testing the structural model.

## 4. Finding and discussion

### 4.1 Measurement model evaluation

This section presents the results of the measurement model evaluation, focusing on the reliability and validity of each construct used in the study. Four main constructs were assessed: Networking Capability, Transformational Organization, Digital Adoption, and Port Performance. To ensure the robustness of the measurement instruments, several key statistical criteria were evaluated, including outer loading, Cronbach’s Alpha, Composite Reliability (rho_A), Composite Reliability (rho_c), and Average Variance Extracted (AVE). Table 1 below shows the results of validity and reliability testing in this study.

**Table 1.** Measurement model evaluation.

Variable	Indicator	Loading	Cronbach alpha	Composite reliability (rho_a)	Composite reliability (rho_a)	AVE
Networking Capability (X1)	X1.1	0.746	0.874	0.891	0.900	0.531
	X1.2	0.806				
	X1.3	0.788				
	X1.4	0.647				
	X1.5	0.738				
	X1.6	0.658				
	X1.7	0.774				
	X1.8	0.653				
Transformational Organization (X2)	X2.1	0.764	0.707	0.725	0.819	0.533
	X2.2	0.605				
	X2.3	0.744				
	X2.4	0.792				
Digital Adoption (Y1)	Y1.1	0.878	0.926	0.928	0.944	0.771
	Y1.2	0.853				
	Y1.3	0.890				
	Y1.4	0.889				
	Y1.5	0.880				
Port Performance (Y2)	Y2.1	0.842	0.876	0.878	0.910	0.671
	Y2.2	0.860				
	Y2.3	0.852				
	Y2.4	0.776				
	Y2.5	0.759				

Based on Table 1, the evaluation of the measurement model begins with an assessment of indicator validity through outer loading values. In a reflective measurement model, the recommended outer loading threshold is at least 0.70 to ensure that the indicators adequately reflect the underlying construct, while indicators with loadings below 0.70 may be considered for elimination if they adversely affect the reliability and validity of the construct. ^63^ The results indicate that most indicators meet this criterion. However, indicators X1.4, X1.6, X1.8, and X2.2 do not satisfy the recommended threshold. Referring to the guidelines for evaluating reflective measurement models, ^63^ this study conducts instrument purification by removing indicators that fail to meet the minimum outer loading criterion, while still considering conceptual relevance and content validity of the constructs. The final results after removing the indicators that do not meet the criteria are presented in Table 2.

**Table 2.** Measurement model evaluation (after the removal of unqualified indicators).

Variable	Indicator	Loading	Cronbach alpha	Composite reliability (rho_a)	Composite reliability (rho_a)	AVE
Networking Capability (X1)	X1.1	0.790	0.840	0.847	0.887	0.610
	X1.2	0.825				
	X1.3	0.815				
	X1.5	0.707				
	X1.7	0.764				
Transformational Organization (X2)	X2.1	0.758	0.702	0.714	0.834	0.626
	X2.3	0.773				
	X2.4	0.841				
Digital Adoption (Y1)	Y1.1	0.878	0.926	0.928	0.944	0.771
	Y1.2	0.853				
	Y1.3	0.890				
	Y1.4	0.889				
	Y1.5	0.880				
Port Performance (Y2)	Y2.1	0.842	0.876	0.878	0.910	0.671
	Y2.2	0.860				
	Y2.3	0.852				
	Y2.4	0.776				
	Y2.5	0.759				

After the purification process, the results presented in Table 2 show that all retained indicators have outer loading values ranging from 0.707 to 0.890, indicating that they generally meet the minimum threshold of 0.70 and approach the reference value of 0.708. This suggests that indicator reliability is adequate for all constructs examined. The next step involves assessing internal consistency and convergent validity at the construct level. Hair et al. ^64^ explain that internal reliability can be evaluated using Cronbach’s alpha and composite reliability, with general guidelines indicating that values between 0.60 and 0.70 are acceptable for exploratory research, values between 0.70 and 0.90 indicate good reliability, and excessively high values may signal indicator redundancy. ^64^ Convergent validity is subsequently assessed using the average variance extracted (AVE), with a minimum recommended threshold of 0.50. ^64^ The results in Table 2 indicate that all constructs exhibit Cronbach’s alpha values ranging from 0.702 to 0.926, composite reliability (rho_c) values ranging from 0.834 to 0.944, and AVE values ranging from 0.610 to 0.771. Therefore, following the removal of indicators that did not meet the outer loading criteria, all constructs in the measurement model demonstrate adequate internal consistency and strong convergent validity, confirming their suitability for testing the structural model in the subsequent stage.

Based on Table 3, discriminant validity is evaluated using the Fornell and Larcker criterion, which states that the square root of the AVE on the diagonal should be greater than the inter-construct correlations in the corresponding row and column. ^65, 66^ The results show that the square root of the AVE for X1 is 0.781, which is greater than its correlations with X2 (0.702), Y1 (0.474), and Y2 (0.537). A similar pattern is also observed for X2, Y1, and Y2, where each diagonal value (0.791; 0.878; 0.819) is higher than the correlations with other constructs. These findings indicate that each construct explains the variance of its own indicators better than the variance it shares with other constructs, thus confirming that discriminant validity is established.

**Table 3.** Fornell and Larcker criterion.

	X1	X2	Y1	Y2
**X1**	0,781			
**X2**	0,702	0,791		
**Y1**	0,474	0,491	0,878	
**Y2**	0,537	0,475	0,535	0,819

Next, Table 4 presents the results of the multicollinearity assessment in the inner model using VIF values. In PLS-SEM reporting, VIF is used to ensure that excessive collinearity does not occur among predictor constructs. VIF values below 3 indicate that there is no multicollinearity issue among the constructs. ^64^ Based on Table 4, the VIF values range from 1.000 to 1.973, indicating that the structural model does not encounter multicollinearity problems, and the estimation of path coefficients can be interpreted with an adequate level of reliability.

**Table 4.** VIF inner model.

	VIF
**X1 -> Y1**	1.973
**X2 -> Y1**	1.973
**Y1 -> Y2**	1.000

### 4.2 Hypothesis testing

Based on Table 5, the results of the direct effects testing show that all proposed hypotheses are empirically supported at the conventional level of significance. First, Networking Capability (X1) has a positive effect on Digital Adoption (Y1), with a path coefficient of 0.255 and a p-value of 0.049. This finding indicates that the stronger a port’s networking capability, the higher the organization’s propensity to adopt digital technologies and systems. Second, Transformational Organization (X2) also has a positive and significant effect on Digital Adoption (Y1), with a path coefficient of 0.312 and a p-value of 0.008. This result shows that transformational organizational characteristics, such as the ability to undertake adaptive internal renewal and change, contribute meaningfully to accelerating digital adoption in the port environment. Third, Digital Adoption (Y1) is shown to have a positive and highly significant effect on Port Performance (Y2), with a path coefficient of 0.535 and a p-value of 0.000. The magnitude of this coefficient indicates that digital adoption is an important determinant of improved port performance, thereby strengthening the argument that digitalization in port operational and service processes plays a direct role in driving organizational performance.

**Table 5.** Direct effect.

	Variable		Path coefficient	P-value	Result
	Predictor	Response			
H1	Networking Capability (X1)	Digital Adoption (Y1)	0.255	0.049	Supported
H2	Transformational Organization (X2)	Digital Adoption (Y1)	0.312	0.008	Supported
H3	Digital Adoption (Y1)	Port Performance (Y2)	0.535	0.000	Supported

Based on Table 6, the indirect effects testing was conducted to assess the mediating role of Digital Adoption (Y1) in the relationship between the independent variables and Port Performance (Y2). The analysis results indicate that the indirect effect of Networking Capability (X1) on Port Performance (Y2) through Digital Adoption (Y1) has a path coefficient of 0.136 with a p-value of 0.069. This p-value exceeds the 0.05 significance level; therefore, hypothesis H4 is not empirically supported. In contrast, the results show that Transformational Organization (X2) has a significant indirect effect on Port Performance (Y2) through Digital Adoption (Y1), with a path coefficient of 0.167 and a p-value of 0.011. This finding supports hypothesis H5 and indicates that Digital Adoption serves as a significant mediator in the relationship between transformational organizational characteristics and port performance. These results imply that an organization’s capability to undertake internal change, renewal, and transformation fosters more effective digital adoption, which in turn positively contributes to improved port performance.

**Table 6.** Indirect effect.

	Variable			Path coefficient	P-value	Result
	Predictor	Mediation	Response			
H4	Networking Capability (X1)	Digital Adoption (Y1)	Port Performance (Y2)	0.136	0.069	Not Supported
H5	Transformational Organization (X2)		Port Performance (Y2)	0.167	0.011	Supported

### 4.3 Discussion

The results of hypothesis testing show that networking capability has a significant effect on digital adoption, thus supporting H1. This finding indicates that the port’s ability to build, manage, and utilize networks with various external stakeholders plays an important role in driving digital technology adoption. The stronger the organization’s networking capability, the greater its ability to effectively adopt digital technology in its operational and strategic activities.

These findings are in line with the study by Waty et al., ^27^ which shows that networking capabilities have a significant effect on digital adoption and business agility. The study confirms that organizations with strong networking capabilities are better able to adopt new technologies quickly and effectively because networks expand access to the latest technological innovations and accelerate knowledge exchange with business partners. These results are also consistent with Al Halbusi et al., ^28^ who found that networking capabilities, particularly through social media networks, have a significant effect on social media adoption. The study emphasizes that customer engagement and the effectiveness of social networks strengthen the use of digital platforms as business tools. From the perspective of Dynamic Capability Theory, ^29^ networking capability can be understood as a dynamic capability that helps organizations respond to the demands of digitalization.

The test results show that transformational organizations have a significant effect on digital adoption, thus supporting H2. These findings confirm that port organizations with transformational characteristics, as reflected in visionary leadership, innovative culture, and employee engagement, tend to be more capable of effectively adopting digital technology. Theoretically, these results are consistent with the view that transformational leaders are able to build a strong vision, inspire motivation, and create a work environment that encourages employees to accept change and challenge outdated practices that are no longer relevant. ^9, 31^ This is important in digital transformation initiatives, which generally face resistance due to uncertainty and disruption of established work routines. ^32^

The empirical findings of this study are in line with the study by Kraft et al., ^37^ which shows that organizational transformation facilitates the adoption of digital tools such as management software and online platforms. These results are also consistent with Bunjak et al., ^38^ who emphasize that transformational leadership and shared leadership play an important role in encouraging the adoption of information technology innovations by employees through the creation of a work environment that supports innovation, learning, and change management. Furthermore, Zahra et al. ^39^ highlight that organizational transformation supported by strong leadership and an innovative culture is a key factor in accelerating digital adoption in the industrial sector. The alignment of these research findings with previous studies reinforces the argument that organizational transformationalism acts as a key enabler for digital adoption. From the perspective of Dynamic Capability Theory, ^29^ organizational transformation reflects the ability to continuously adapt, reconfigure, and develop internal resources and capabilities in response to environmental changes.

The results of this study also found that digital adoption has a significant effect on port performance, thus supporting H3. These findings indicate that the higher the level of digital technology adoption in port operations, the better the port performance achieved. This relatively strong influence confirms that digitalization is not merely an operational complement, but a strategic factor that directly contributes to improving port efficiency, service quality, and competitiveness. These findings are in line with Subasinghe, ^42^ who, through a systematic literature review, concluded that port digitalization plays an important role in improving competitiveness and operational sustainability. The use of technologies such as automation, the Internet of Things, and artificial intelligence has been proven to help ports manage the flow of goods and information more efficiently, reduce operational costs, and strengthen collaboration between stakeholders. These results are also consistent with Jiang et al., ^43^ who found that port-based information integration has a significant effect on port performance through improved operational efficiency, coordination between actors, and data-driven decision-making. Furthermore, Othman et al. ^44^ emphasized that the implementation of smart port practices supports sustainable performance improvement by strengthening economic, social, and environmental dimensions.

From the Resource-Based View and Dynamic Capability perspectives, these findings reinforce the view that digital adoption constitutes a strategic mechanism for optimizing and reconfiguring port operational resources to align with dynamic environmental demands. ^29, 45^ Digital technologies enable ports to improve resource-use efficiency, enhance workflows, and strengthen coordination within a complex port ecosystem. However, these results also indicate that the benefits of digital adoption for performance are not automatic, but rather depend heavily on the readiness of an organization’s internal capabilities and the external environmental context faced by the port. ^46^

## 5. Conclusion, limitation and future research

This study concludes that improvements in port performance are not only determined by traditional operational factors, but increasingly depend on an organization’s ability to build strategic capabilities and convert them into effective digital practices. The results show that networking capability and transformational organization play important roles as determinants of digital adoption, as both expand ports’ access to knowledge, information, and technological resources from the external environment, while simultaneously shaping internal readiness through visionary leadership, an innovative culture, organizational learning, and more adaptive structures toward change. Furthermore, digital adoption is shown to have a significant effect on port performance through improvements in process efficiency and reliability, service acceleration, strengthened coordination among stakeholders, and enhanced service quality that contributes to competitiveness. Moreover, this study confirms the mediating role of digital adoption in the relationship between networking capability and port performance as well as between transformational organization and port performance.

Although offering important theoretical and practical contributions, this study is not without limitations. First, this research is contextually limited to Indonesian container ports. Second, this study uses a cross-sectional design, which captures organizational dynamics and performance perceptions at a single point in time. This limits the ability to assess the temporal or causal evolution of digital adoption and its long-term effects on port performance. Therefore, we suggest that future research take a longitudinal approach to deepen existing insights. In addition, this study relies on perceptual data collected through self-report questionnaires from port management representatives. Although such data is valuable for capturing internal assessments of capabilities and performance, it may introduce subjectivity or response bias. Future studies could strengthen the findings by incorporating objective performance metrics, such as throughput records, waiting times, or digital system usage logs.

Future research should expand the model to diverse port contexts, including international comparative studies, to validate and refine the findings. Researchers may also consider examining additional organizational factors, such as innovation culture, knowledge management practices, or IT governance structures, as complementary antecedents to digital transformation. Furthermore, qualitative or mixed methods approaches can enrich the understanding of the mechanisms behind successful digital integration, particularly in complex institutional settings. By addressing these limitations, future studies can build a more comprehensive and context-sensitive body of knowledge on digital transformation in the port and logistics sector.

## Ethical approval statement

This study received ethical approval from the Ethics Committee, Faculty of Administrative Sciences, Universitas Brawijaya (Brawijaya University), Indonesia. Ethical approval was granted under Ethical Approval Letter No. 09910/UN10.F0301/B/PP/2025. The study was conducted in accordance with applicable institutional and national ethical guidelines for research involving human participants.

## Informed consent statement

All participants were provided with clear information about the study objectives, procedures, potential risks and benefits, confidentiality protections, and their right to decline or withdraw at any time without penalty. Written informed consent was obtained from all participants prior to data collection. Participants’ identities were anonymized, and data were reported in aggregate to prevent individual identification.

## Data Availability

The datasets generated and/or analyzed during the current study are not publicly available due to ethical restrictions and confidentiality commitments to participants. However, the data are available from the corresponding author upon reasonable request and subject to approval by the relevant ethics requirements. Requests should be submitted via email to the corresponding author at: buyung@student.ub.ac.id. Access may be granted for research purposes only, and requesters may be required to sign a data use agreement and to ensure that data are stored securely and used in compliance with participant confidentiality. **Underlying Data** Figshare: *Supporting Research Data “Enhancing Port Performance through Digital Transformation: The Role of Networking Capability and Transformational Organization in Indonesian Container Ports”* https://doi.org/10.6084/m9.figshare.31015432 ^67^ The project contains the following underlying data:
• Questionnaire Details (List of variables and item that used in this study) Data are available under the terms of the Creative Commons Attribution 4.0 International license (CC-BY 4.0).
