# Supplementary material for: Enhancing Port Performance through Digital Transformation: The Role of Networking Capability and Organizational transformation in Indonesian Container Ports
Source: F1000Res. 2026 Apr 29;15:87. Originally published 2026 Jan 20. [Version 3] doi: 10.12688/f1000research.176261.3 (PMC13033128; doi:10.12688/f1000research.176261.3)
Supplement: Peer review history

# Reviewer report: Reviewer response for version 3

**DOI:** 10.5256/f1000research.198827.r480269

**Published:** 2026-05-06

**Authors:** Benny Hutahayan

**Affiliations:** 1 University of Brawijaya, Malang, East Java, Indonesia

The author has revised the manuscript based on the suggestions I provided. Good luck

Is the work clearly and accurately presented and does it cite the current literature?

Yes

If applicable, is the statistical analysis and its interpretation appropriate?

Yes

Are all the source data underlying the results available to ensure full reproducibility?

Yes

Is the study design appropriate and is the work technically sound?

Yes

Are the conclusions drawn adequately supported by the results?

Yes

Are sufficient details of methods and analysis provided to allow replication by others?

Partly

Reviewer Expertise:

Business Administration, Human Resources Management, Law, and Governance

I confirm that I have read this submission and believe that I have an appropriate level of expertise to confirm that it is of an acceptable scientific standard.

# Reviewer report: Reviewer response for version 3

**DOI:** 10.5256/f1000research.198827.r480268

**Published:** 2026-05-04

**Authors:** Sofwan Farisyi

**Affiliations:** 1 Trisakti University, West Jakarta, Jakarta, Indonesia

I have no further comments to make.

Is the work clearly and accurately presented and does it cite the current literature?

Yes

If applicable, is the statistical analysis and its interpretation appropriate?

Yes

Are all the source data underlying the results available to ensure full reproducibility?

Yes

Is the study design appropriate and is the work technically sound?

Partly

Are the conclusions drawn adequately supported by the results?

Partly

Are sufficient details of methods and analysis provided to allow replication by others?

Partly

Reviewer Expertise:

Expertise in shipping and port. I am Head of Indonesian National Shipowners' Association

I confirm that I have read this submission and believe that I have an appropriate level of expertise to confirm that it is of an acceptable scientific standard.

# Reviewer report: Reviewer response for version 2

**DOI:** 10.5256/f1000research.197091.r467160

**Published:** 2026-03-30

**Authors:** Benny Hutahayan

**Affiliations:** 1 University of Brawijaya, Malang, East Java, Indonesia

I have a small revision for you to add please include the CMB test results. Overall, your manuscript is ready for indexing.

Is the work clearly and accurately presented and does it cite the current literature?

Yes

If applicable, is the statistical analysis and its interpretation appropriate?

Yes

Are all the source data underlying the results available to ensure full reproducibility?

Yes

Is the study design appropriate and is the work technically sound?

Yes

Are the conclusions drawn adequately supported by the results?

Yes

Are sufficient details of methods and analysis provided to allow replication by others?

Partly

Reviewer Expertise:

Business Administration, Human Resources Management, Law, and Governance

I confirm that I have read this submission and believe that I have an appropriate level of expertise to confirm that it is of an acceptable scientific standard.

# Response

**Published:** 2026-04-13

**Authors:** Buyung Pramitra

**Affiliations:** Not Applicable, Malang, East Java, Indonesia

Dear Reviewer, Thank you for your suggestion. I have added the CMB test results to my manuscript in the latest revision. Thank you.

# Reviewer report: Reviewer response for version 2

**DOI:** 10.5256/f1000research.197091.r467632

**Published:** 2026-03-28

**Authors:** Sofwan Farisyi

**Affiliations:** 1 Trisakti University, West Jakarta, Jakarta, Indonesia

1. In the abstract, the authors should clearly articulate the rationale for conducting this study. Specifically, it is important to highlight the research gap identified in previous studies and to emphasize the novelty or unique contribution of this research.

 2. The manuscript should maintain consistency in terminology by using either “organizational transformation” or “transformational organization” uniformly throughout the text.

 3. In the introduction, the authors should provide a more detailed elaboration of the research gap, particularly in comparison with studies conducted in developed countries, as well as clearly differentiate this study from prior research.

 4. In the literature review, the authors are encouraged to provide a thematic synthesis and comparative analysis of prior studies, clearly indicating which findings report significant positive, significant negative, or non-significant results.

 5. In Section 2.4 (Mediating Role of Digital Adoption), there is an inconsistency in the numbering of Hypotheses 3 and 4 when compared to Figure 1. These should be revised to Hypotheses 4 and 5 to ensure alignment with the figure.

 6. In the conclusion, the statement *“Moreover, this study confirms the mediating role of digital adoption in the relationship between networking capability and port performance as well as between organizational transformation and port performance”* appears to be inconsistent with the reported results, which indicate that *Hypothesis H4 is not empirically supported*. This discrepancy should be clarified to ensure consistency between the findings and the conclusions.

Is the work clearly and accurately presented and does it cite the current literature?

Yes

If applicable, is the statistical analysis and its interpretation appropriate?

Yes

Are all the source data underlying the results available to ensure full reproducibility?

Yes

Is the study design appropriate and is the work technically sound?

Partly

Are the conclusions drawn adequately supported by the results?

Partly

Are sufficient details of methods and analysis provided to allow replication by others?

Partly

Reviewer Expertise:

Expertise in shipping and port. I am Head of Indonesian National Shipowners' Association

I confirm that I have read this submission and believe that I have an appropriate level of expertise to confirm that it is of an acceptable scientific standard, however I have significant reservations, as outlined above.

# Response

**Published:** 2026-04-13

**Authors:** Buyung Pramitra

**Affiliations:** Not Applicable, Malang, East Java, Indonesia

Thank you for your feedback. Your revisions are invaluable to my manuscript. I have revised my manuscript based on your suggestions.

1. We have revised the abstract by adding a more explicit explanation of the research gap.
2. We have reviewed the entire manuscript and standardized the use of terminology to ensure consistency.
3. We have expanded the discussion of the research gap in the Introduction section.
4. We understand that thematic synthesis and comparative analysis will strengthen the theoretical foundation of the research. In the revised version, we have improved the literature review section.
5. We have revised the numbering of the hypotheses in Subsection 2.4 to align with Figure 1. In the revised version, Hypotheses 3 and 4 have been renumbered as Hypotheses 4 and 5 to ensure consistency between the text and the research model in the figure.
6. We agree that the statement is not fully consistent with the empirical results of the study. Therefore, we have revised the Conclusion section to align with the reported findings.  

# Reviewer report: Reviewer response for version 2

**DOI:** 10.5256/f1000research.197091.r467159

**Published:** 2026-03-20

**Authors:** Faisal Binsar

**Affiliations:** 1 Universitas Muhammadiyah Berau, Tandjungredeb, Kalimantan Timur, Indonesia; 2 Management Department Binus Online Learning, Binus University, Universitas Muhammadiyah Berau, Jakarta, DKI Jakarta, Indonesia

The authors have made considerable efforts to address the reviewer comments, and the manuscript has improved in several aspects, particularly in the clarification of constructs, hypotheses, and methodological description.

 However, there are still several corrections that need to be addressed:

 1. Change H3 in the hypothesis statement to H4.

 H3: Digital adoption mediates the relationship between networking capability and port performance.

 2. Change H4 in the hypothesis statement to H5.

 H4: Digital adoption mediates the relationship between organizational transformation and port performance.

 3. In "Figure 1. Conceptual Framework," the dotted lines for H4 and H5 should touch the circle for the "Digital Adoption" variable as a mediator. Currently, H4 and H5 show a direct relationship between the variables. The legends in "Explanations" are interchanged.

Is the work clearly and accurately presented and does it cite the current literature?

Partly

If applicable, is the statistical analysis and its interpretation appropriate?

Yes

Are all the source data underlying the results available to ensure full reproducibility?

Partly

Is the study design appropriate and is the work technically sound?

Partly

Are the conclusions drawn adequately supported by the results?

Yes

Are sufficient details of methods and analysis provided to allow replication by others?

Partly

Reviewer Expertise:

Digital transformation, Organizational performance, Leadership, Entrepreneur and Innovation

I confirm that I have read this submission and believe that I have an appropriate level of expertise to confirm that it is of an acceptable scientific standard.

# Reviewer report: Reviewer response for version 1

**DOI:** 10.5256/f1000research.194303.r460975

**Published:** 2026-02-28

**Authors:** Benny Hutahayan

**Affiliations:** 1 University of Brawijaya, Malang, East Java, Indonesia

Dear Author

 I appreciate you for writing a good manuscript. The manuscript has been written coherently in accordance with scientific guidelines. However, I have a few suggestions to improve the quality of your manuscript.

 1. First, the background has been written clearly and coherently. However, the gap in this study is quite simple and needs to be expanded to further improve the quality of the manuscript.

 2. This study has also mentioned the literature review and hypothesis development quite well. A few improvements are also needed for the formulation of hypotheses, there are two hypotheses 3 (H3). Please double-check.

 3. Figure 1 needs to be corrected, especially in hypotheses 4 and 5. You should draw lines from networking capability and organizational transformation, not from digital adoption.

 4. This study uses cross-sectional data, therefore it needs to test for common method bias (CMB).

 5. The results and discussion have been explained quite well. However, I did not find any discussion related to H4 and H5, so please add them.

 6. The manuscript would be stronger if it included the research instrument questionnaire items.

Is the work clearly and accurately presented and does it cite the current literature?

Yes

If applicable, is the statistical analysis and its interpretation appropriate?

Yes

Are all the source data underlying the results available to ensure full reproducibility?

Yes

Is the study design appropriate and is the work technically sound?

Yes

Are the conclusions drawn adequately supported by the results?

Yes

Are sufficient details of methods and analysis provided to allow replication by others?

Partly

Reviewer Expertise:

Business Administration, Human Resources Management, Law, and Governance

I confirm that I have read this submission and believe that I have an appropriate level of expertise to confirm that it is of an acceptable scientific standard, however I have significant reservations, as outlined above.

# Reviewer report: Reviewer response for version 1

**DOI:** 10.5256/f1000research.194303.r455641

**Published:** 2026-02-14

**Authors:** Faisal Binsar

**Affiliations:** 1 Universitas Muhammadiyah Berau, Tandjungredeb, Kalimantan Timur, Indonesia; 2 Management Department Binus Online Learning, Binus University, Universitas Muhammadiyah Berau, Jakarta, DKI Jakarta, Indonesia

1. This model is relatively linear and has been widely researched in other contexts (SMEs, banking, healthcare, manufacturing). Capability → Digital Adoption → Performance is a very common model in the digital transformation literature. Please clearly articulate what theoretical advancement it offers beyond contextual replication.

 2. Transformational Organization is unclear. Is it transformational leadership? Organizational transformation? Organizational culture? Please analyze further in comment 12.

 3. Revise the statements for all hypotheses. Hypothesis statements should not contain "significant."

 4. There are two H3 hypotheses in the manuscript, and no H5.

 5. Figure 1. Conceptual framework still contains errors and deficiencies. Review the direct and indirect effect lines. The diagram should accurately reflect all hypothesized direct and indirect relationships.

 6. How many informants were selected for each port? Explain.

 7. When was the survey and data collection conducted? Through what means?

 8. Present respondent profile/demographic data, including port category, operating period, informant level, etc.

 9. Explain why unqualified indicators occurred and had to be removed. Please justify whether the deletion was theory-driven or purely statistical.

 10. Two categories of ports: specifically handling containers and multipurpose ports that also handle containers. Based on the findings of this study, explain which category has the most significant networking capability, transformational organization, and digital adoption on port performance. Explain why.

 11. Explain why Networking Capability has no impact on Port Performance through Digital Adoption. However, in organizations in other sectors, it is very significant, such as hospitals or health care centers in the healthcare sector. Please provide a theoretical explanation for this unexpected finding.

 12. Some references appear to be non-peer-reviewed or introductory sources. Please prioritize high-impact, peer-reviewed international journals. International journal standards are usually more selective. To strengthen and generalize this study, the following relevant literature is recommended:

 a. The role of digital adoption capability on hospital performance in Indonesia moderated by environmental dynamism **(Refer 1)**

 b. Determinants of Digital Adoption Capability for Service Performance in Indonesian Hospitals: A Conceptual Model ( **Refer 2)**

Is the work clearly and accurately presented and does it cite the current literature?

Partly

If applicable, is the statistical analysis and its interpretation appropriate?

Yes

Are all the source data underlying the results available to ensure full reproducibility?

Partly

Is the study design appropriate and is the work technically sound?

Partly

Are the conclusions drawn adequately supported by the results?

Yes

Are sufficient details of methods and analysis provided to allow replication by others?

Partly

Reviewer Expertise:

Digital transformation, Organizational performance, Leadership, Entrepreneur and Innovation

I confirm that I have read this submission and believe that I have an appropriate level of expertise to confirm that it is of an acceptable scientific standard, however I have significant reservations, as outlined above.

## References

[1] : The role of digital adoption capability on hospital performance in Indonesia moderated by environmental dynamism. *Journal of Health Organization and Management* .2025;39(1) : 10.1108/JHOM-04-2024-0130 1-21 10.1108/JHOM-04-2024-0130 41126338

[2] : Determinants of Digital Adoption Capability for Service Performance in Indonesian Hospitals: A Conceptual Model. *https://doi.org/10.33168/JSMS.2024.0212* .2024;

# Response

**Published:** 2026-02-27

**Authors:** Buyung Pramitra

**Affiliations:** Not Applicable, Malang, East Java, Indonesia

Thank you for the valuable suggestions and comments. The reviewer’s feedback has been very helpful in improving the manuscript, particularly in clarifying the theoretical contribution, strengthening conceptual consistency, and improving methodological reporting transparency. Below, we provide our point by point responses.

 1) We revised the Introduction to clarify the study’s theoretical contribution beyond contextual replication, particularly by emphasizing digital adoption as a mechanism linking organizational factors to port performance.

 2) We clarified the construct terminology and consistently corrected the ambiguous term throughout the manuscript, changing transformational organization to organizational transformation.

 3) We revised all hypothesis statements to align with academic conventions by removing the term significant and stating the hypothesized relationships appropriately.

 4) We corrected the hypothesis numbering so that it is sequential and consistent (H1 to H5) throughout the manuscript.

 5) We reviewed and revised Figure 1 to ensure consistency with all hypothesized direct and indirect relationships.

 6) We added a clarification in the Methods section that each of the 103 ports was represented by one key informant at the managerial level.

 7) We added the data collection period in the Methods section.

 8) We added a respondent demographic table along with its interpretation to improve transparency of sample characteristics.

 9) We added a clearer justification for indicator deletion in the measurement model.

 10. We clarified that this study was designed from the outset to test the hypothesized relationships in the pooled sample rather than compare effects across port categories. A category comparison would require a different analytical design and is beyond the scope of the current study.

 11) We strengthened the discussion of the finding that Networking Capability has no impact on Port Performance through Digital Adoption by adding a theoretical and contextual explanation, and by emphasizing that the generalizability of results may differ across sectors due to sector specific operational and institutional characteristics.

 12) We added and updated references by prioritizing relevant peer reviewed international journal sources.
